# Design of a High-Throughput Real-Time PCR System for Detection of Bovine Respiratory and Enteric Pathogens

**DOI:** 10.3389/fvets.2021.677993

**Published:** 2021-06-24

**Authors:** Nicole B. Goecke, Bodil H. Nielsen, Mette B. Petersen, Lars E. Larsen

**Affiliations:** ^1^Centre for Diagnostics, Technical University of Denmark, Lyngby, Denmark; ^2^Department of Veterinary and Animal Sciences, University of Copenhagen, Copenhagen, Denmark; ^3^Department of Animal Science, Aarhus University, Aarhus, Denmark; ^4^Department of Veterinary Clinical Sciences, University of Copenhagen, Copenhagen, Denmark

**Keywords:** high-throughput, real-time PCR, viruses, bacteria, bovine pathogens, prevalence

## Abstract

Bovine respiratory and enteric diseases have a profound negative impact on animal, health, welfare, and productivity. A vast number of viruses and bacteria are associated with the diseases. Pathogen detection using real-time PCR (rtPCR) assays performed on traditional rtPCR platforms are costly and time consuming and by that limit the use of diagnostics in bovine medicine. To diminish these limitations, we have developed a high-throughput rtPCR system (BioMark HD; Fluidigm) for simultaneous detection of the 11 most important respiratory and enteric viral and bacterial pathogens. The sensitivity and specificity of the rtPCR assays on the high-throughput platform was comparable with that of the traditional rtPCR platform. Pools consisting of positive and negative individual field samples were tested in the high-throughput rtPCR system in order to investigate the effect of an individual sample in a pool. The pool tests showed that irrespective of the size of the pool, a high-range positive individual sample had a high influence on the cycle quantification value of the pool compared with the influence of a low-range positive individual sample. To validate the test on field samples, 2,393 nasal swab and 2,379 fecal samples were tested on the high-throughput rtPCR system as pools in order to determine the occurrence of the 11 pathogens in 100 Danish herds (83 dairy and 17 veal herds). In the dairy calves, *Pasteurella multocida* (38.4%), rotavirus A (27.4%), *Mycoplasma* spp. (26.2%), and *Trueperella pyogenes* (25.5%) were the most prevalent pathogens, while *P. multocida* (71.4%), *Mycoplasma* spp. (58.9%), *Mannheimia haemolytica* (53.6%), and *Mycoplasma bovis* (42.9%) were the most often detected pathogens in the veal calves. The established high-throughput system provides new possibilities for analysis of bovine samples, since the system enables testing of multiple samples for the presence of different pathogens in the same analysis test even with reduced costs and turnover time.

## Introduction

Bovine respiratory and enteric diseases have a profound negative impact on animal, health, welfare, and productivity. The two major calf disease syndromes are bovine respiratory disease (BRD) and bovine enteric disease (BED) which are multifactorial diseases associated with presence of a range of pathogens, environmental factors, stress conditions, and health and immunological status of the animal. Bovine respiratory disease and BED can have substantive economic consequence due to reduced productivity, increased mortality, and/or morbidity, as well as decreased animal welfare and increased use of antibiotics (1–3).

Bovine respiratory disease is most severe in calves between 2 weeks and 6 months of age. A wide range of viruses and bacteria are involved in BRD, including bovine adenovirus, bovine coronavirus (BCoV), bovine herpesvirus 1 (BHV1), bovine parainfluenza virus type 3, bovine respiratory syncytial virus (BRSV), bovine viral diarrhea virus (BVDV), *Mannheimia haemolytica, Pasteurella multocida, Histophilus somni*, and *Mycoplasma bovis* (4–6). The viruses BHV1 and BVDV have been eradicated in several countries, including Denmark (7). In addition to the abovementioned viruses, influenza D virus (IDV), bovine rhinitis A virus, and bovine torovirus (BToV) have recently been shown to be involved in BRD (8–10). Furthermore, the bacterium *Trueperella pyogenes* has also been associated with BRD (6). Development of severe respiratory signs often involves a primary viral infection followed by a secondary bacterial infection (1, 11, 12). The progression of BRD is believed to be related to suppression of the immune system, allowing for inflammation and damage of the respiratory tissue, which in severe cases can lead to pneumonia or even death (13).

Bovine enteric disease is often associated with diarrhea, which is one of the most economically costly disorders in the calves industry due to weight loss and deaths of young animals (3). Multiple viruses, bacteria, and parasites have been identified as the causative agents of diarrhea. The most important infectious agents are BCoV, rotavirus A (RVA), BVDV, *Escherichia coli* F5 (K99+), *Salmonella* spp., *Clostridium perfringens, Cryptosporidium parvum*, and *Eimeria* spp. Several of these pathogens are associated with diarrhea within a particular age group (14–17). Furthermore, viruses such as bovine norovirus, bovine enterovirus, rotavirus B and C, BToV, and nebovirus have also been shown to be potential diarrhea-causing pathogens (14, 18–21). Each of these pathogens can cause disease individually, but mixed infections are also commonly seen, which often lead to more severe disease (14, 22).

Since a vast number of viral and bacterial pathogens are involved in both BRD and BED, it is essential to have a highly specific and sensitive diagnostic method for rapid identification of the causative pathogens. A variety of laboratory tests, including culture and molecular methods, have been described and these methods all have their benefits and limitations in regard to sensitivity, specificity, predictive values, speed, and costs (23). During recent years, a range of multiplex real-time PCR (rtPCR) tests targeting pathogens involved in BRD and/or BED have been developed (24–28). The multiplex rtPCR test allows for simultaneous analysis of three–five pathogens in a single sample. However, the number of available targets that can be tested in one run is limited because multiplex rtPfoCR is based on traditional rtPCR platforms, which have a limited number of detection channels. A general disadvantage of the common used tests is the high costs. Therefore, pooling of individual samples can be beneficial and cost effective especially as it requires no additional equipment or materials (29). Pooling can be favorable in screening and surveillance programs, and if information at the individual sample level is required, subsequent individual tests can be performed if the pooled sample is positive.

In order to diminish the limitations of the traditional rtPCR platforms, we previously have established high-throughput rtPCR systems for detection and screening of respiratory and enteric viral and bacterial porcine pathogens (30, 31) and for detection and differentiation of influenza A viruses circulating in Danish pigs (32). The high-throughput rtPCR platform BioMark HD (Fluidigm, South San Francisco, CA) and the dynamic array (DA) integrated fluidic circuit (IFC) nanofluidic chip have been utilized. Different DA IFC chips exist which can combine either 48 samples with 48 assays (48.48DA), 96 samples with 96 assays (96.96DA), 192 samples with 24 assays (192.24DA), or 24 samples with 192 assays (24.192DA), resulting in 2,304, 9,216 or 4,608 individual reactions, respectively. The rtPCR reactions are carried out in the DA IFC chip, which contains microfluidic networks that automatically combine the samples and rtPCR reagents in the reaction chambers. Furthermore, the high-throughput platform has also been used as a screening and detection tool for tick-borne and food- and water-borne pathogens (33, 34).

In the present paper, we describe the design, optimization, validation, and use of a similar high-throughput rtPCR system consisting of 11 rtPCR assays targeting 11 respiratory and enteric viral and bacterial bovine pathogens known to be involved in BRD and BED. The purpose of the high-throughput rtPCR system was to develop a system that can function as a rapid screening and detection tool suitable for the detection of disease-causing pathogen(s) within calf herds. Furthermore, pools consisting of different numbers of positive and negative individual field samples were tested in order to investigate the effect of the individual samples in a pool. Lastly, the occurrence of the 11 respiratory and enteric viral and bacterial pathogens in Danish calves was evaluated by using the developed high-throughput rtPCR system.

## Materials and Methods

### Samples and Sampling

Known positive samples (controls) were used for optimization and initial validation of the high-throughput rtPCR system and the associated rtPCR assays. The positive controls consisted of pure bacterial cultures, cell culture lysates (viruses), and synthesized plasmids coding for the specific PCR targets. Initially, the positive controls were tested by culturing and/or by established and validated PCR assays and/or sequencing. The positive controls were obtained from the routine diagnostic laboratory at the Centre for Diagnostics, Technical University of Denmark (DTU). Furthermore, field samples (nasal swab and fecal samples) collected from Danish calves were used for validation of the high-throughput rtPCR system. Nasal swab samples were collected by inserting a sterile cotton swab (Technical University of Denmark, Lyngby, Denmark) approximately 8–10 cm into one nostril and turning the swab around for a few seconds. No prior cleaning of the nostril was performed. Immediately after, the swabs were placed and stored in 1.5 ml phosphate-buffered saline (PBS). Fecal samples were collected from each calf by gathering the feces in a 10-ml tube when the calf was expelling feces from the rectum. If the calf did not defecated spontaneously, a finger was inserted into the rectum and defecation was stimulated by gentle manipulation of the intestinal wall. The samples were kept refrigerated (approximately 5°C) for up to 48 h prior to shipment, and they were sent in a box containing freezer packs to the Centre for Diagnostics, DTU, where the samples were stored at −80°C until nucleic acid extraction. Prior to extraction, a 10% dilution in PBS was made for each of the individual fecal samples by weighing 0.1 g of the feces and adding PBS. The nasal swab samples were vortexed to transfer the biological material to PBS. The nasal swab and fecal samples were analyzed as individual samples and/or as pools. Before the nucleic acid extraction, the samples were pooled based on herd and age group with five to 10 samples per pool. The 10% feces dilutions were pooled with equal volume (μl) of each individual sample. The nasal swab samples were also pooled with equal volume (μl) of each individual sample. For the samples, which were analyzed both individually and in a pool, the sample material used for both analyses originated from the same original sample for the nasal swab samples. For the feces samples, the sample material used came from the same 10% dilution of the original sample.

For the field study, 4,772 field samples (2,393 nasal swab and 2,379 fecal samples) were collected from 100 Danish, intensive, commercial herds (83 dairy and 17 veal herds). The veal herds were rosé veal producers that produce meat from calves fed on a diet without restriction of iron intake. The rosé veal calves are slaughtered when they are between 8 and 12 months old. Sampling was done in the winter period from September to April in 2018 and 2019. The samples were collected from three age groups in the dairy herds (0–10 days, 3 weeks, 3 months) and two age groups in the veal herds (2 weeks after arrival and at 3 months of age). In the first and second age groups, calves were primarily kept in single pens. In the two oldest age groups, calves were kept indoor in groups. Feeding regimes differed according to local management. Typically, the calves were milk fed for 8–12 weeks. In 14 cases, it was not possible to obtain a fecal sample, as the calf did not defecate and the rectum was empty. Therefore, the number of fecal samples differed from the number of nasal swab samples.

### Nucleic Acid Extraction

RNA and DNA were extracted from the nasal swab samples using the extraction robot QIAcube HT (QIAGEN, Hilden, Germany) and the Cador Pathogen 96 QIAcube HT kit (QIAGEN) using the manufacturer's instructions. Before nucleic acid extraction, nasal swab samples were prepared by centrifuging 400 μl of each individual sample or pool for 5 min at 9,000 × *g* at room temperature (15–25°C), and 200 μl of the supernatant was subsequently used for extraction. Positive and negative (nuclease-free water; Amresco, Cleveland, OH) controls were included in each extraction. The nucleic acids were stored at −80°C until further analysis.

RNA and DNA were extracted from 10% fecal dilutions of the individual samples or from pools consisting of the 10% fecal dilutions using the extraction robot QIAcube HT (QIAGEN) and the Cador Pathogen 96 QIAcube HT kit (QIAGEN). Prior to nucleic acid extraction, one 5-mm steel bead was added to each sample or pool and the samples or pools were homogenized in a TissueLyser II (QIAGEN) for 20 s at 15 Hz. The homogenate was centrifuged for 90 s at 6,700 × *g*, and 200 μl of the supernatant was used for extraction. Positive and negative (nuclease-free water; Amresco) controls were included in each extraction. The nucleic acid extractions were stored at −80°C until further analysis.

### Primer and Probe Design

Eleven rtPCR assays targeting respiratory and enteric viral and bacterial pathogens were established (Table 1). The primer and probe sequences were copied either from previously published assays or designed in this study. Some of the published primer and probe sequences were modified to improve the specificity or to adapt to the selected PCR conditions. New primer and probe sequences were designed based on alignments containing sequences of the target gene for the selected pathogens. The sequences were retrieved from GenBank (35) and aligned using CLC Main Workbench version 8.0 (QIAGEN). The specificity of the oligonucleotides were tested *in silico* using nucleotide BLAST search (36), and the melting temperature and basic properties were approximated using OligoCalc (37). The oligonucleotides were purchased from Eurofins Genomics (Ebersberg, Germany).

**Table 1:** Viruses and bacteria, assay names, and primer and probe sequences used for detection of viruses and bacteria.

**Pathogen**	**Target gene**	**Name**	**Sequence (5^**′**^-3^**′**^)**	**Length (bp)**	**Reference**
BRSV	F	BRSV-F-485F	AAGGGTCAAACATCTGCTTAACTAG	85	Hakhverdyan et al. (66)
		BRSV-F-569R	TCTGCCTGWGGGAAAAAAG		
		BRSV F Taqman-546	FAM-AGAGCCTGCATTRTCACAATACCACCCA-BHQ1		
BCoV	M	BCoV-F	GTTGGTGGAGTTTCAACCCAG	90	F, R, and P (modified): Decaro et al. (65)
		BCoV-R	GGTAGTCCTCAATTATCGGCC		
		BCoV-P	FAM-CATCCTTCCCTTCATATCTATACACATC-BHQ1		
*E. coli* F5		*E. coli* F5-F	GAGGTCAATGGTAATCGTACATC	117	This study
		*E. coli* F5-R	CGCTAGGCAGTCAYTACTGC		
		*E. coli* F5-P	FAM-GATCTTGGGCAGGCTGCTATTAGTGGT-BHQ1		
*H. somni*	16S rRNA	HS-F	GAAGATACTGACGCTCGAGT	115	F and P: this study; R: Angen et al. (64)
		HS-R	TTCGGGCACCAAGTRTTCA		
		HS-P	FAM-TCCCCAAATCGACATCGTTTACAGCGTG-BHQ1		
IDV	PB1	IDV-F	GCTGTTTGCAAGTTGATGGG	136	Hause et al. (50)
		IDV-R	TGAAAGCAGGTAACTCCAAGG		
		IDV-P	FAM-TTCAGGCAAGCACCCGTAGGATT-BHQ1		
*M. haemolytica*	*sodA*	M. hae-F	GCCGTTGTTTCAACCGCTAAC	100	This study
		M. hae-R	CGTGTTCCCAAACGTCTAAGAC		
		M. hae-P	FAM-TCGGATAGCCTGAAACGCCTGCCAC-BHQ1		
*M. bovis*	*oppD*	PMB996-F	TCAAGGAACCCCACCAGAT	71	Sachse et al. (68)
		PMB1066-R	AGGCAAAGTCATTTCTAGGTGCAA		
		Mbovis1016	FAM-TGGCAAACTTACCTATCGGTGACCCT-TAMRA		
*Mycoplasma* spp.	16S rRNA	Mycoplasma-F	GATCCTGGCTCAGGATGAAC	103	This study
		Mycoplasma-R	CGTTGAGTACGTGTTACTCAC		
		Mycoplasma-P	FAM-GGCTGTGTGCCTAATACATGCATGTCG-BHQ1		
*P. multocida*	*kmt1*	PM-ny-F	GACTACCGACAAGCCCACTC	125	F and R: this study; P: Goecke et al. (30)
		PM-ny-R	CTATCCGCTATTTACCCAGTGG		
		PM-P	FAM-GTGCGAATGAACCGATTGCCGCG- BHQ1		
RVA	NSP3	Rota A-F	ACCATCTACACATGACCCTC	84	F and P: Pang et al. (67); R: this study
		Rota A-ny-R	CACATAACGCCCCTATAGCC		
		Rota A-P	FAM-ATGAGCACAATAGTTAAAAGCTAACACTGTCAA-TAMRA		
*T. pyogenes*	plo-Pyolysin	*T. pyogenes*-F	CATCAACAATCCCACGAAGAG	98	F (modified) and R: Kishimoto et al. (25); P: this study
		*T. pyogenes*-R	TTGCAGCATGGTCAGGATAC		
		*T. pyogenes*-P	FAM-CCGTGACTCAAGGACTGAACGGCCT-BHQ1		

### Traditional rtPCR Platform

Initially, the sensitivity and specificity of the rtPCR assays were validated on the Rotor-Gene Q rtPCR platform (QIAGEN) using 10-fold serial dilutions of the positive controls. For the rtPCR assays targeting RNA viruses, AgPath-ID one-step RT-PCR reagents kit (Applied Biosystems, Foster City, CA) was used with a final reaction volume of 15 μl. The PCR mix consisted of 7.5 μl RT-PCR buffer (2 ×), 0.45 μl of each primer (10 μM), 0.45 μl probe (10 μM), 0.6 μl RT-PCR enzyme mix (25 ×), 3.55 μl nuclease-free water, and 2 μl RNA. The PCR reactions were run at the following thermal cycling conditions: 45°C for 20 min, 95°C for 10 min, followed by 45 cycles of 94°C for 15 s, and 60°C for 45 s.

For the rtPCR assays targeting DNA viruses and bacteria, JumpStart Taq ready mix (Sigma-Aldrich, St. Louis, MO) was used with a final reaction volume of 25 μl. The PCR mix contained 12.5 μl JumpStart Taq ready mix (2 ×), 0.75 μl of each primer (10 μM), 0.2 μl probe (30 μM), 3.5 μl MgCl_2_ (25 mM), 4.3 μl nuclease-free water, and 3 μl DNA. The PCR reactions were tested at the following thermal cycle conditions: 94°C for 2 min, followed by 40 cycles of 94°C for 15 s, and 60°C for 60 s.

Data, including quantification cycle (Cq) values and amplification curves, obtained from the abovementioned PCR reactions were analyzed using Rotor-Gene series software version 2.3.1 (QIAGEN) with the following parameter adjustments: dynamic tube normalization, on; noise slope correction, on; ignore first cycle; outlier removal, 10%; and Cq fixed, 0.01. All reactions, samples, and positive and negative (nuclease-free water; Amresco) controls were run in duplicates. For each of the rtPCR assays, a standard curve was constructed from the Cq values. The amplification efficiency was calculated based on the slope of the standard curve, as previously described (38).

### Reverse Transcription and Preamplification Prior to High-Throughput rtPCR

For RNA targets, one-tube combined reverse-transcription and preamplifications were performed in a final volume of 15 μl using AgPath-ID one-step RT-PCR reagents kit (Applied Biosystems); 7.5 μl RT-PCR buffer (2 ×), 0.75 μl of 200 nM primer mix (containing the different sets of primers (20 μM each) listed in Table 1), 0.6 μl random hexamer (50 μM), 0.6 μl RT-PCR enzyme mix (25 ×), 2.55 nuclease-free water, and 3 μl RNA were mixed. The PCR was performed on a T3 Thermocycler (Biometra, Fredensborg, Denmark) with the following thermal cycle conditions: 45°C for 20 min, 95°C for 10 min, followed by 24 cycles at 94°C for 15 s, and 60°C for 45 s. The preamplified complementary DNA (cDNA) was stored at −20°C.

For DNA targets, preamplification were performed using TaqMan PreAmp master mix (Applied Biosystems) in a final volume of 10 μl containing 5 μl master mix, 2.5 μl of 200 nM primer mix [containing the different sets of primers (20 μM each) listed in Table 1], and 2.5 μl DNA. Preamplification was performed on a T3 Thermocycler (Biometra) with the following thermal cycle conditions: 95°C for 10 min, followed by 14 cycles at 95°C for 15 s, and 60°C for 4 min. The preamplified DNA was stored at −20°C until testing.

### High-Throughput rtPCR

For the rtPCR analysis, the high-throughput rtPCR platform BioMark HD (Fluidigm) and the BioMark 192.24 DA IFC chip (Fluidigm) were used. For each sample, a 4-μl sample mix containing 2 μl TaqMan gene expression master mix (2 ×) (Applied Biosystems), 0.2 μl sample loading reagent (20 ×) (Fluidigm), and 1.8 μl preamplified sample was prepared. For each assay, a 4-μl assay mix containing 2 μl assay loading reagent (2 ×) (Fluidigm) and 2 μl primer-probe stock (final concentration: 16 μM primers and 5 μM probe) was prepared. Three-microliter sample mix and 3 μl assay mix were loaded into the respective inlets of the 192.24 DA IFC chip. The 192.24.DA IFC chip was placed in the IFC controller RX (Fluidigm) for loading and mixing for approximately 30 min and then subject to thermal cycling in the high-throughput rtPCR instrument BioMark HD (Fluidigm) with the following cycle conditions: 50°C for 2 min, 95°C for 10 min, followed by 40 cycles of 95°C for 15 s and 60°C for 60 s. Samples were tested in single reactions, and the assays were performed in duplicates. In each 192.24 DA IFC chip run, positive and negative (nuclease-free water; Amresco) PCR and extraction controls were included. Data, including Cq values and amplification curves, obtained on the BioMark system, were analyzed using the Fluidigm Real-Time PCR Analysis software version 4.5.2 (Fluidigm).

### Assessment of the Sensitivity, Specificity and Application of the rtPCR Assays

Initially, the rtPCR assays were validated on the Rotor-Gene Q (QAGEN) and BioMark HD (192.24 DA IFC) (Fluidigm) platforms by running 10-fold serial dilutions for each of the positive controls in duplicates in order to analyze the sensitivity and amplification efficiency on the two platforms. For each of the rtPCR assays, a standard curve was constructed from the Cq values. The amplification efficiency was calculated based on the slope of the standard, curve as previously described (38).

To assess the specificity of the rtPCR assays on the high-throughput rtPCR platform (BioMark HD; Fluidigm), the positive controls were initially tested, followed by testing of 32 field samples (19 nasal swab and 13 fecal samples). Six field samples positive for either *E. coli* F5 or *M. haemolytica* were selected for Sanger sequencing in order to verify the specificity of the rtPCR assays. Prior to sequencing, the selected samples were PCR amplified on the Rotor-Gene Q platform (QIAGEN), and the PCR products were purified using the High Pure PCR Product Purification kit (Roche, Basel, Switzerland) according to the manufacturer's instructions. The samples were sequenced at LGC Genomics GmbH (Berlin, Germany). The obtained sequences were assembled and analyzed using CLC Main Workbench version 8.0 (QIAGEN). The analyzed sequences were aligned to published sequences using the database NCBI BLAST (36).

To evaluate the repeatability of the rtPCR assays on the high-throughput rtPCR platform (BioMark HD; Fluidigm), the positive controls were tested on 13 separated 192.24 DA IFC chip runs and the outcomes were compared along the chip runs.

The application of the high-throughput rtPCR platform (BioMark HD; Fludigm) and 192.24 DA IFC chip was validated by testing 4,772 field samples, which were pooled in 980 pools (491 pools of nasal swab and 489 pools of fecal samples).

### Assessment of Test of Pooled Samples Contra Test of Individual Samples

In order to compare test of pooled samples with test of individual samples, a pilot study was performed. Three different setups were made for five selected assays (*Mycoplasma* spp., *M. bovis, H. somni, T. pyogenes*, and RVA) using field samples selected based on the results obtained in the high-throughput rtPCR analysis.

In the first and second setup, the effect of increasing the number of positive samples and decreasing the number of negative samples within a pool was tested. In the first setup, the pools consisted of 10 samples with a varying number of positive and negative samples. The pools were made with the following distribution of positive and negative samples; 1:9, 2:8, 3:7, 4:6, 5:5, 6:4, 7:3, 8:2, and 9:1 (number of positive samples:number of negative samples). Similarly, the second setup tested pools consisting of five samples instead of 10 samples. In the third setup, the effect of increasing the number of negative samples within a pool containing one positive sample was tested. Here, the pools consisted of one positive sample and an increasing number of negative samples with the following structure 1:1, 1:2, 1:3, 1:4, 1:5, 1:6, 1:7, and 1:8 (number of positive samples:number of negative samples). The constructed pools from the three setups were analyzed on the high-throughput rtPCR system, as described above.

## Results

### Sensitivity and Amplification Efficiency of the rtPCR Assays

To evaluate the sensitivity and amplification efficiency of the rtPCR assays, 10-fold serial dilutions of the positive controls were tested on the Rotor-Gene Q (QIAGEN) and BioMark HD (Fluidigm) platforms. Standard curves were constructed using mean Cq values from duplicate 10-fold serial dilutions, in which the efficiency was calculated for each of the rtPCR assays (Table 2).

**Table 2:** Sensitivity and amplification efficiency of rtPCR assays on the Rotor-Gene Q and BioMark platforms.

**Assays**																						
**Dilution**	**BRSV**		**BCoV**		***E. coli*** **F5**		***H. somni***		**IDV**		***M. haemolytica***		***M. bovis***		***Mycoplasma*** **spp**.		***P. multocida***		**RVA**		***T. pyogenes***	
	**Rotor-gene**	**BioMark**	**Rotor-gene**	**BioMark**	**Rotor-gene**	**BioMark**	**Rotor-gene**	**BioMark**	**Rotor-gene**	**BioMark**	**Rotor-gene**	**BioMark**	**Rotor-gene**	**BioMark**	**Rotor-gene**	**BioMark**	**Rotor-gene**	**BioMark**	**Rotor-gene**	**BioMark**	**Rotor-gene**	**BioMark**
10	12.7	NA	18.6	12.6	12.6	3.3	9.0	(3.3)	23.6	11.0	11.0	(2.5)	12.6	(2.5)	13.0	(3.4)	10.9	(3.5)	18.4	6.3	(11)	(2.5)
10^−1^	15.5	13.0	22.0	16.5	15.5	7.7	11.4	(3.7)	26.5	14.3	14.1	2.8	15.9	3.3	15.9	3.9	13.8	3.9	21.6	8.9	12.8	(2.5)
10^−2^	18.6	18.8	25.4	19.5	19.6	13.0	14.5	2.5	30.1	17.4	17.7	5.8	19.1	6.0	19.5	7.6	17.7	6.7	25.4	12.4	15.5	2.5
10^−3^	22.4	21.0	28.6	21.4	23.6	15.8	17.6	6.2	34.1	21.4	21.1	9.3	22.6	9.1	22.9	10.9	21.1	10.3	28.1	15.7	19.4	6.0
10^−4^	25.4	25.4	32.3	24.7	28.6	17.6	20.9	9.0	–	–	24.6	12.4	26.2	12.8	26.3	15.0	24.6	14.1	32.0	18.5	23.6	9.9
10^−5^	28.2	27.9	35.4	–	(33.1)	20.9	24.5	12.6	–	–	28.0	16.0	29.5	15.9	29.9	17.3	27.8	16.7	34.9	23.7	27.0	13.3
10^−6^	32.3	–	–	–	(37.8)	23.9	28.7	16.8	–	–	31.1	19.0	33.3	18.7	32.9	19.9	31.0	19.8	–	26.0	30.3	17.4
10^−7^	(34.4)	–	–	–	–	–	32.2	19.3	–	–	–	22.2	–	21.2	36.3	–	–	(24.6)	–	–	33.2	–
10^−8^	–	–	–	–	–	–	–	–	–	–	–	–	–	(23.7)	–	–	–	–	–	–	–	–
Efficiency	1.03	0.79	0.98	1.18	0.78	1.02	0.99	0.97	0.92	1.03	0.97	1.01	0.95	1.09	0.98	1.09	0.97	1.03	0.99	0.99	0.92	0.83

*Numbers: Cq values. “–” negative sample; NA, sample was not analyzed. Number in parentheses is not included in the efficiency calculation*.

The sensitivity of the rtPCR assays was in the range of 3–7 log_10_, and the results were identical or differed by one log_10_ between the two platforms (Table 2). Similarly, the dynamic range of the rtPCR assays was either identical or differed by one log_10_. Furthermore, the amplification efficiency of the rtPCR assays was comparable for the Rotor-Gene Q (QIAGEN) and the BioMark HD (Fluidigm) platforms and was 78–103% and 79–118%, respectively. For some of the rtPCR assays (*H. somni, T. pyogenes*), the undiluted and the first diluted sample were excluded from the calculation of efficiency of the BioMark HD (Fluidigm) platform because the Cq was too low. This exclusion resulted in a shorter dynamic range for these rtPCR assays when run on the BioMark HD (Fluidigm) platform compared with the Rotor-Gene Q (QIAGEN) run.

The Cq values obtained by the BioMark HD (Fluidigm) platform were allocated into three categories; high-range positive (Cq value ≤ 13), mid-range positive (Cq value 14–20), and low-range positive (Cq value ≥21).

### Test of the Specificity of the rtPCR Assays

**Figure 1:**
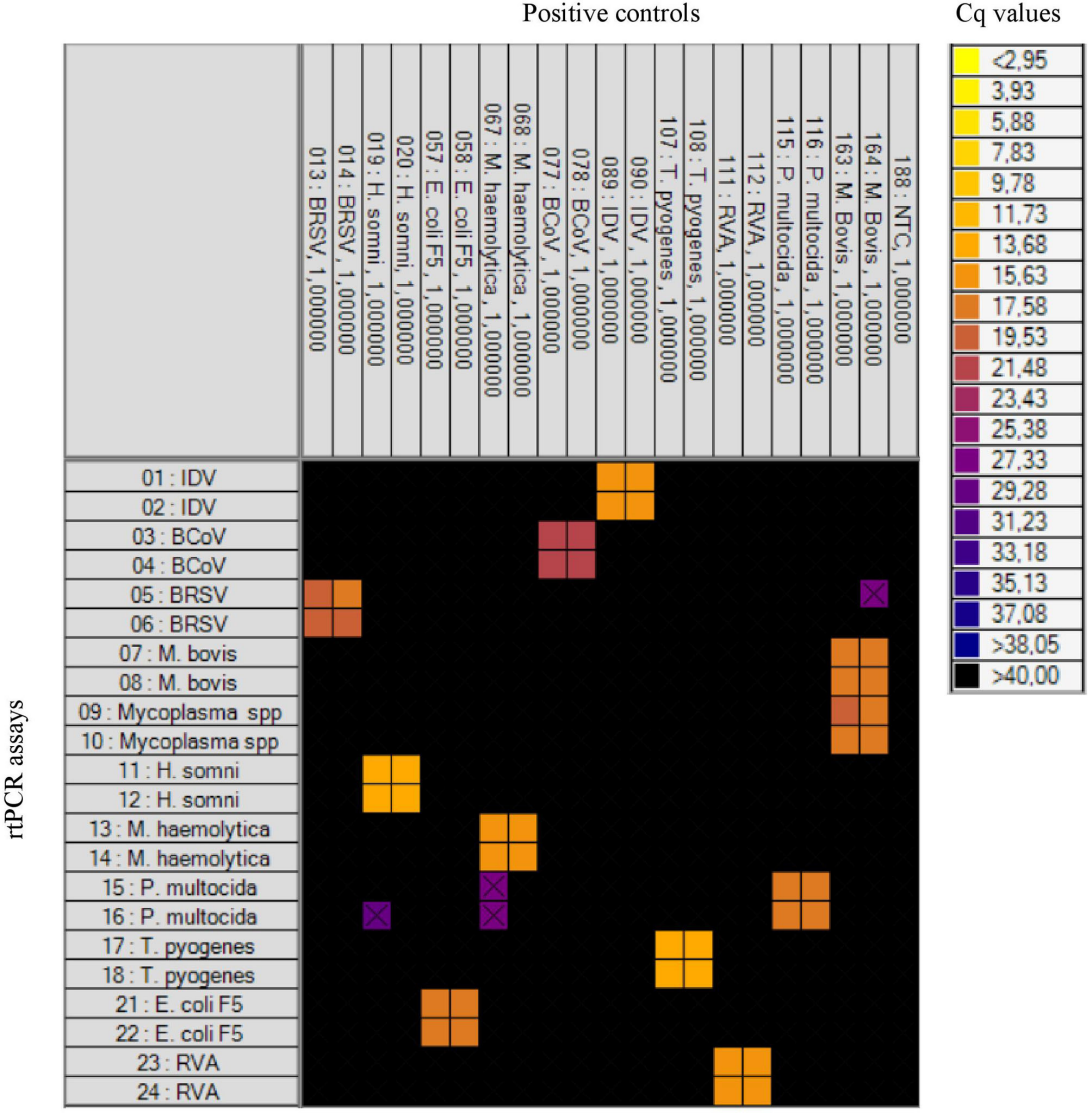
Heat map showing the specificity of the rtPCR assays on the high-throughput rtPCR system by testing known positive controls. To the left: rtPCR assays (Table 1). At the top: the positive controls and a no-template control (NTC). Each square corresponds to a single rtPCR reaction. Cq values for each reaction are indicated by color; the corresponding color scale is presented in the legend on the right. A black square is considered a negative result. A black X is shown if the amplification curve deviates too much from an ideal amplification curve.

Initially, the specificity of the rtPCR assays was tested on the BioMark HD (Fluidigm) platform and the 192.24 DA IFC chip by testing the positive controls for each pathogen in all assays. For each rtPCR assay, the specificity was assessed based on the Cq value and the corresponding amplification curve obtained from the respective positive control. Positive results were obtained in the rtPCR assay specific for the correct positive control sample only—that is, no cross-reaction to any of the other positive control samples was detected (Figure 1). Furthermore, 32 field samples (nasal swab and fecal samples) were tested on the high-throughput system in order to investigate the performance on field samples (Table 3). Six of these field samples, which tested positive for either *E. coli* F5 or *M. haemolytica*, were selected for Sanger sequencing (Table 4). The obtained sequences were aligned to previously published sequences using the database NCBI BLAST (36). Eight sequences showed 100% identity to the two field samples positive for *E. coli* F5 (samples 2 and 4). For three out of four *M. haemolytica*-positive field samples, 89 sequences showed 100% identity to these (samples 15, 19, and 20), while for the last field sample (sample 21), the 89 sequences showed 98.7–99.0% identity. The accession numbers of the published sequences which showed the highest identity to the sequences obtained in this study are listed in Table 4.

**Table 3:** Feces and nasal swab samples analyzed on the high-throughput rtPCR system.

**Sample**	**Material**	**BRSV**	**BCoV**	***M. bovis***	***Mycoplasma* spp**.	***M. haemolytica***	***H. somni***	**IDV**	***P. multocida***	**RVA**	***E. coli F5***
1	Feces		–	–	–					–	29.0
2	Feces		–	–	–					17.0	22.4
3	Feces		–	–	–					6.5	–
4	Feces		–	–	–					–	21.5
5	Feces		–	–	–					–	27.6
6	Feces		–	–	–					–	–
7	Feces		–	–	–					–	–
8	Feces		–	–	–					–	–
9	Feces		–	–	–					–	–
10	Feces		19.0	–	–					–	–
11	Feces		–	–	–					–	–
12	Feces		–	–	–					–	–
13	Feces		–	–	–					–	–
14	Nasal swab	–	–	–	24.0	–	–	–	–		
15	Nasal swab	–	–	–	23.4	22.4	–	–	17.2		
16	Nasal swab	–	–	–	–	–	–	–	–		
17	Nasal swab	–	–	–	–	–	–	–	–		
18	Nasal swab	–	–	–	–	–	–	–	–		
19	Nasal swab	–	–	17.4	13.1	15.0	–	–	18.3		
20	Nasal swab	–	–	15.9	15.5	18.1	–	–	17.5		
21	Nasal swab	–	–	–	15.2	16.8	18.6	–	21.0		
22	Nasal swab	–	26.2	16.5	12.8	13.8	–	–	18.9		
23	Nasal swab	–	–	25.9	11.3	14.7	19.5	–	19.9		
24	Nasal swab	–	–	–	NA	–	20.0	–	18.2		
25	Nasal swab	–	–	–	NA	17.2	–	–	18.6		
26	Nasal swab	–	–	–	NA	20.7	14.1	–	19.7		
27	Nasal swab	–	–	–	NA	–	20.5	–	17.7		
28	Nasal swab	–	–	–	NA	–	–	–	21.3		
29	Nasal swab	–	–	–	NA	–	–	–	–		
30	Nasal swab	–	–	–	NA	22.5	19.1	–	–		
31	Nasal swab	–	–	–	NA	21.3	23.2	–	–		
32	Nasal swab	–	–	–	NA	20.66	–	–	27.4		

*Numbers: Cq values.“–” negative result; NA, sample was not analyzed. Gray cell: analysis not relevant*.

**Table 4:** Samples sequenced by Sanger sequencing.

**Sample**	**Pathogen**	**BLAST results—accession no**.
2	*E. coli* F5	MH916617, KR870316, KR606337, KP054295, JX987524, GU951525, FJ864678, M35282 (100% identity)
4	*E. coli* F5	MH916617, KR870316, KR606337, KP054295, JX987524, GU951525, FJ864678, M35282 (100% identity)
15	*M. haemolytica*	CP017484-17552, CP026857-58, CP029638, LS483299, CP023043-44, CP023046-47, CP006957, CP004752-53, CP011098-99, CP006619, CP005972-74, CP005383, AY702551, AY702512 (100% identity)
19	*M. haemolytica*	CP017484-17552, CP026857-58, CP029638, LS483299, CP023043-44, CP023046-47, CP006957, CP004752-53, CP011098-99, CP006619, CP005972-74, CP005383, AY702551, AY702512 (100% identity)
20	*M. haemolytica*	CP017484-17552, CP026857-58, CP029638, LS483299, CP023043-44, CP023046-47, CP006957, CP004752-53, CP011098-99, CP006619, CP005972-74, CP005383, AY702551, AY702512 (100% identity)
21	*M. haemolytica*	CP017484-17552, CP026857-58, CP029638, LS483299, CP023043-44, CP023046-47, CP006957, CP004752-53, CP011098-99, CP006619, CP005972-74, CP005383, AY702551, AY702512 (98.96%−98.73% identity)

*Comparison of percentage identity with already published sequences*.

### Test of Repeatability of the rtPCR Assays on the High-Throughput rtPCR Platform

The repeatability of the rtPCR assays on the high-throughput rtPCR platform (BioMark HD) was evaluated by testing the positive controls in 13 separate chip runs. The mean Cq value and standard deviation (SD) were calculated for each of the positive controls, and the SD was found to be between ±0.35 and 1.00 for all the positive control samples (Table 5).

**Table 5:** Test of repeatability of the rtPCR assays on the high-throughput rtPCR platform.

**Positive control**	**No. of repeats**	**Mean Cq value**	**Standard deviation (±)**
BRSV	13	21.7	1.00
BCoV	13	21.0	0.92
*E. coli* F5	13	13.7	0.97
*H. somni*	13	16.5	0.48
IDV	13	19.0	0.83
*M. haemolytica*	13	16.2	0.44
*M. bovis*	13	18.8	0.70
*Mycoplasma* spp.	13	20.2	0.60
*P. multocida*	13	14.0	0.35
RVA	13	16.3	0.76
*T. pyogenes*	13	12.5	0.46

### Comparative Testing of Pooled and Individual Samples

The results of the three different pool setups are shown for each pathogen in Supplementary Tables 1A–E. In general, the results from the first and second setup showed that irrespective of the size of the pool, an individual sample with a high-range positive Cq value had a higher influence on the Cq value of the pool compared with the influence of an individual sample with a low-range positive Cq value.

For all five pathogens in the first setup, there was a decrease in the Cq value of the first pool (1:9) to the last pool (9:1), meaning that the pool became increasingly positive. However, the degree of decrease was varied between the different pathogens. For *Mycoplasma* spp., *H. somni*, and RVA there was a decrease of 5.3–6.4 Cq values, while for *M. bovis* and *T. pyogenes*, the decrease in Cq was 1.6 and 0.3, respectively. In the second setup, the Cq value of the pool decreased (became more positive) with increasing numbers of positive samples for four of the pathogens. For *H. somni*, the Cq values for two of the pools (3:2 and 4:1) were 17.0 and 17.1, respectively, and therefore a decrease in the Cq value of the pool was not observed. In the third setup, in which an increasing number of negative individual samples were added to a pool containing one positive sample, the pool became less positive (higher Cq value) as the number of negative samples increased. For all of the five pathogens, the pool was only made to dilution 1:8 due to a limited amount of available negative samples. For the RVA pool, the Cq value increased from 8.8 (pool 1:1) to 16.4 (pool 1:8), which was more than expected in relation to a 10-fold dilution in theory should increase the Cq value with a value of 3.3. The positive sample in the RVA pool had a high-range positive Cq value (8.3) compared with the positive samples in the *Mycoplasma* spp., *M. bovis, H. somni*, and *T. pyogenes* pools, which had a Cq value between 16.4 and 17.9.

### Occurrences of Respiratory and Enteric Pathogens in Danish Calves

**Figure 2:**
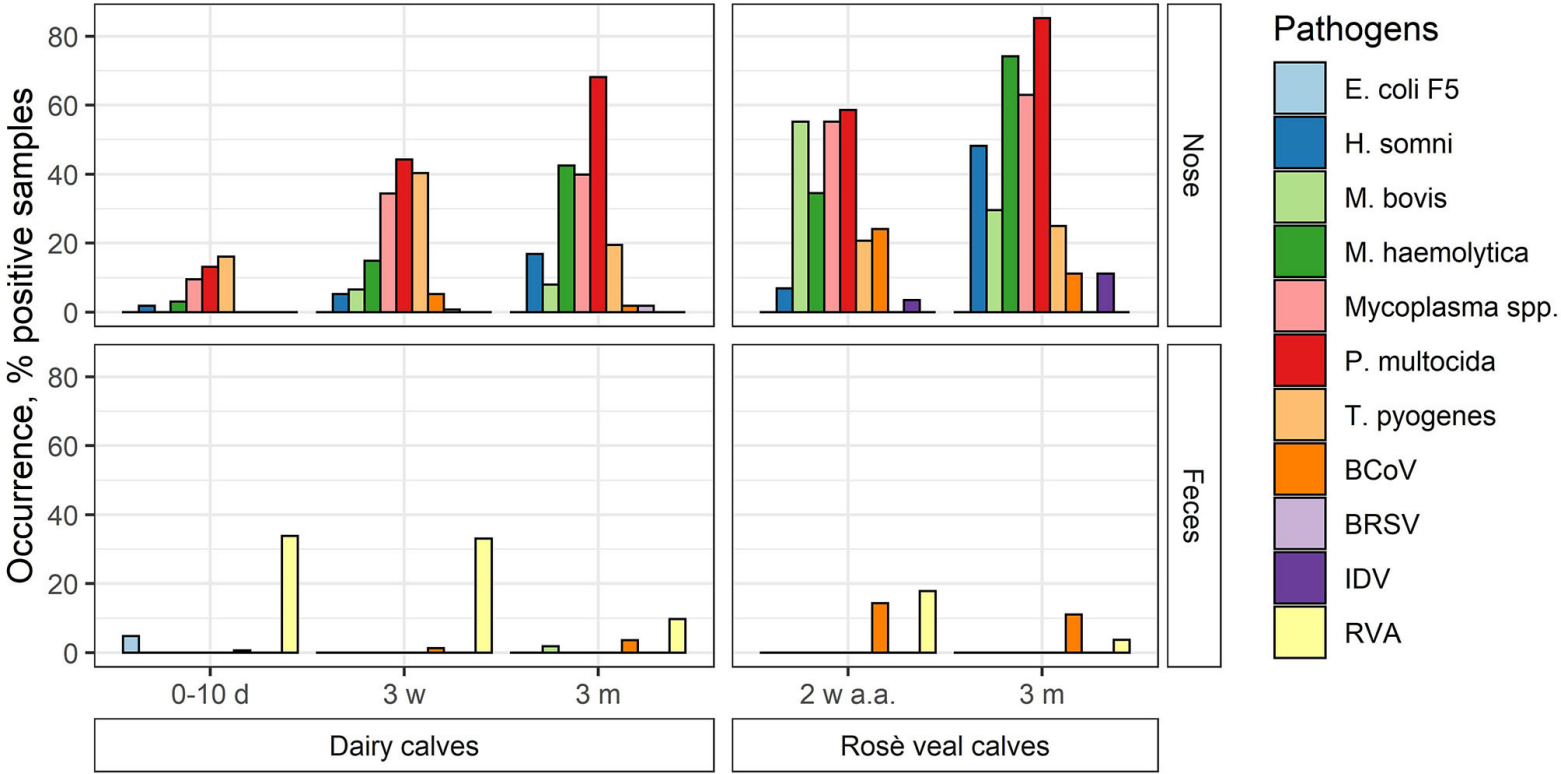
The occurrence of respiratory and enteric pathogens (viruses and bacteria) in nasal swab (nose) and feces pools made of samples taken from Danish dairy and rosé veal calves at different ages (d, day; w, week; m, month; a.a., after arrival) in percentage.

The occurrence of the different pathogens in Danish calves was evaluated by testing 980 pools of nasal swab and feces samples on the high-throughput rtPCR system. The samples were collected from dairy and veal calves at different ages from 100 Danish herds (83 dairy and 17 veal herds). In total, 491 nasal swab pools and 489 feces pools were analyzed and the overall occurrence of each pathogen was calculated (Figure 2; Table 6). Furthermore, the occurrence of each pathogen in each age group on the herd level was calculated as the number of herds with at least one positive pool divided by the total number of herds (Table 7).

**Table 6:** The occurrence of respiratory and enteric pathogens by age group and in total.

	**Number of positive nasal swab pools**							**Number of positive feces pools**						
	**Dairy calves**			**Overall**	**Veal calves**		**Overall**	**Dairy calves**			**Overall**	**Veal calves**		**Overall**
**Pathogens**	**0–10 days**	****~**3 weeks**	****~**3 months**	***N* = 435 (%)**	****~**2 weeks**	****~**3 months**	***N* = 56 (%)**	**0–10 days**	****~**3 weeks**	****~**3 months**	***N* = 434 (%)**	****~**2 weeks**	****~**3 months**	***N* = 55 (%)**
	***N*** **=** **168 (%)**	***N*** **=** **154 (%)**	***N*** **=** **113 (%)**		***N*** **=** **29 (%)**	***N*** **=** **27 (%)**		***N*** **=** **168 (%)**	***N*** **=** **154 (%)**	***N*** **=** **112 (%)**		***N*** **=** **28 (%)**	***N*** **=** **27 (%)**	
BRSV	0 (0)	1 (0.7)	2 (1.8)	3 (0.7)	0 (0)	0 (0)	0 (0)							
BCoV	0 (0)	8 (5.2)	2 (1.8)	10 (2.3)	7 (24.1)	3 (11.1)	10 (17.9)	1 (0.6)	2 (1.3)	4 (3.6)	7 (1.6)	4 (14.3)	3 (11.1)	7 (12.7)
IDV	0 (0)	0 (0)	0 (0)	0 (0)	1 (3.5)	3 (11.1)	4 (7.1)							
*M. bovis*	0 (0)	10 (6.5)	9 (8.0)	19 (4.4)	16 (55.2)	8 (29.6)	24 (42.9)	(0)	(0)	1 (0.9)	1 (0.2)	(0)	(0)	(0)
*Mycoplasma* spp.	16 (9.5)	54 (34.4)	45 (39.8)	114 (26.2)	16 (55.2)	17 (63.0)	33 (58.9)	(0)	(0)	(0)	(0)	(0)	(0)	(0)
*M. haemolytica*	5 (3.0)	23 (14.9)	48 (42.5)	76 (17.5)	10 (34.5)	20 (74.1)	30 (53.6)							
*H. somni*	3 (1.8)	8 (5.2)	19 (16.8)	30 (6.9)	2 (6.9)	13 (48.2)	15 (26.8)							
*P. multocida*	22 (13.1)	68 (44.2)	77 (68.1)	167 (38.4)	17 (58.6)	23 (85.2)	40 (71.4)							
*T. pyogenes*	27 (16.1)	62 (40.3)	22 (19.5)	111 (25.5)	6 (20.7)	7 (25.0)	13 (23.2)							
*E. coli F5*								8 (4.8)	(0)	(0)	8 (1.8)	(0)	(0)	(0)
RVA								57 (33.9)	51 (33.1)	11 (9.8)	119 (27.4)	5 (17.9)	1 (3.7)	6 (10.9)

*Number of positive pools, followed by prevalence (%) in parentheses*.

*N, number of analyzed pools; %, percent of positive pools. Gray cell: analysis not relevant*.

**Table 7:** The occurrence of respiratory and enteric pathogens at herd level by age group.

	**Number of positive nasal swab pools**							**Number of positive feces pools**						
	**Dairy calves**			**Overall^▴^**	**Veal calves**		**Overall^▴^**	**Dairy calves**			**Overall^▴^**	**Veal calves**		**Overall^▴^**
**Pathogens**	**0–10 days**	****~**3 weeks**	****~**3 months**		****~**2 weeks**	****~**3 months**		**0–10 days**	****~**3 weeks**	****~**3 months**		****~**2 weeks**	****~**3 months**	
	***N*** **=** **83 (%)**	***N*** **=** **83 (%)**	***N*** **=** **83 (%)**		***N*** **=** **16 (%)**	***N*** **=** **17 (%)**		***N*** **=** **83 (%)**	***N*** **=** **83 (%)**	***N*** = 82^*^(%)		***N*** = 16^*^ (%)	***N*** **=** **17 (%)**	
BRSV	0 (0)	1 (1.2)	2 (2.4)	2 (2.4)	0 (0)	0 (0)	0 (0)							
BCoV	0 (0)	5 (6.0)	2 (2.4)	6 (7.2)	5 (31.3)	3 (17.7)	6 (35.3)	1 (1.2)	1 (1.2)	4 (4.9)	6 (7.2)	4 (25.0)	3 (17.7)	6 (35.3)
IDV	0 (0)	0 (0)	0 (0)	0 (0)	1 (6.3)	3 (17.7)	4 (23.5)							
*M. bovis*	0 (0)	7 (8.4)	8 (9.6)	13 (15.7)	12 (75.0)	6 (35.3)	13 (76.5)	(0)	(0)	1 (1.2)	1 (1.2)	(0)	(0)	0 (0)
*Mycoplasma* spp.	12 (14.5)	38 (45.8)	37 (44.6)	53 (63.9)	13 (81.3)	13 (76.5)	16 (94.1)	(0)	(0)	(0)	0 (0)	(0)	(0)	0 (0)
*M. haemolytica*	5 (6.0)	17 (20.5)	40 (48.2)	45 (54.2)	8 (50.5)	15 (88.2)	16 (94.1)							
*H. somni*	3 (3.6)	7 (8.4)	14 (16.9)	17 (20.5)	1 (6.3)	8 (47.1)	8 (47.1)							
*P. multocida*	19 (22.9)	43 (51.8)	61 (73.5)	67 (80.7)	14 (87.5)	15 (88.2)	16 (94.1)							
*T. pyogenes*	23 (27.7)	44 (53.0)	22 (26.5)	51 (61.5)	5 (31.3)	5 (29.4)	7 (41.2)							
*E. coli F5*								6 (7.2)	(0)	(0)	6 (7.2)	(0)	(0)	0 (0)
RVA								37 (44.6)	40 (48.2)	11 (13.4)	55 (66.3)	4 (25.0)	1 (5.9)	5 (29.4)

*Number of herds with at least one positive pool in the age group, followed by prevalence (%) in parentheses*.

*N, number of analyzed pools; %, percent of positive pools. Gray cell: analysis not relevant*.

▴ *Across all age groups in the herd*.

* *In one herd, we obtained no fecal samples*.

#### Overall Pathogen Occurrence

In general, the overall occurrence of the respiratory pathogens in the different age groups showed that the bacterial pathogens were more frequent than the viruses both in the dairy and veal calves (Table 6). In the dairy calves, *P. multocida* (38.4%), *Mycoplasma* spp. (26.2%), *T. pyogenes* (25.5%), and *M. haemolytica* (17.5%) were found to be the most prevalent pathogens across all age groups followed by *H. somni* (6.9%) and *M. bovis* (4.4%). For the bacterial pathogens, the highest occurrences were found in the oldest age group except for *T. pyogenes*, which was most frequent in the middle age group. The viral pathogens BCoV and BRSV were present at a very low level (2.3 and 0.7%, respectively), while none of the pools from the dairy calves tested positive for IDV. In the veal calves, more than 50% of the pools were positive for *Mycoplasma* spp., *M. haemolytica*, or *P. multocida*, and the occurrence increased with age. Also, *M. bovis* (42.9%) was frequently detected, while *H. somni* (26.8%) and *T. pyogenes* (23.2%) were less frequently detected. For the viral pathogens, BCoV was the virus with the highest overall occurrence (17.9%) followed by IDV (7.1%), while BRSV was not detected in any pools from the veal calves. Bovine coronavirus was most frequently detected in the youngest age group (24.1%), while IDV was most frequently detected in the oldest age group (11.1%).

The enteric pathogens generally had lower occurrence than the respiratory pathogens. Rotavirus A was the most frequently detected enteric pathogen both in the dairy (27.4%) and veal (10.9%) calves with the highest occurrence in the youngest age groups (17.9%−33.9%). Bovine coronavirus was observed in all age groups and was most prevalent in the veal calves (12.7%). *E. coli* F5 was only detected in the youngest age group in dairy calves (1.8%), and *M. bovis* was only detected in a single pool in the 3-month age group in dairy calves (0.2%).

#### Occurrence of Pathogens at the Herd Level

The occurrence of the pathogens at the herd level in the different age groups is shown in Table 7. At the overall herd level, the majority of the dairy herds had at least one nasal swab pool testing positive for *P. multocida* (67 herds, 80.7%), *Mycoplasma* spp. (53 herds, 63.9%), *T. pyogenes* (51 herds, 61.5%), or *M. haemolytica* (45 herds, 54.2%). Seventeen herds (20.5%) were positive for *H. somni* and 13 herds (15.7%) for *M. bovis*, while BCoV and BRSV were detected in six (7.2%) and two (2.4%) of the dairy herds, respectively. Influenza D virus was not detected in any of the dairy herds. Six dairy herds (7.2%) had at least one feces pool positive for *E. coli* F5 and one herd (1.2%) tested positive for *M. bovis*. Rotavirus A and BCoV were detected in 55 (66.3%) and six (7.2%) herds, respectively.

In the veal herds, the majority of the herds had at least one nasal swab pool testing positive for *P. multocida* (16 herds, 94.1%), *Mycoplasma* spp. (16 herds, 94.1%), *M. haemolytica* (16 herds, 94.1%), or *M. bovis* (13 herds, 76.5%). *H. somni* was detected in eight herds (47.0%) and *T. pyogenes* in seven herds (41.2%). The viruses BCoV and IDV were found in six (35.3%) and four veal herds (23.5%), respectively, while none of the veal herds tested positive for BRSV. Considering the feces pools from veal herds, none of the herds tested positive for *E. coli* F5, *Mycoplasma* spp., or *M. bovis*, while six herds (35.3%) were positive for BCoV, and RVA was detected in five herds (29.4%).

## Discussion

The validation of the high-throughput rtPCR system revealed that it was possible simultaneously to test for 11 respiratory and enteric pathogens, including four viruses and seven bacteria known to be associated with BRD and/or BED. The sensitivity and specificity of the rtPCR assays were evaluated using positive controls tested both on the high-throughput BioMark HD and the Rotor-Gene Q platforms. These analyses revealed that all assays had an acceptable PCR efficiency, and only minor differences in the dynamic range and efficiency were seen between the two platforms. In general, the Cq values obtained on the BioMark HD platform were lower than those of the traditional rtPCR platform. This discrepancy in values is probably due to the preamplification of the samples in the high-throughput setup (39). Preamplification of samples is often required due to the small reaction volumes in the BioMark system, and this step is also recommended from the supplier and other studies using this platform for pathogen detection (30, 33, 40). An important aspect to consider when using the BioMark HD platform is the risk of false-negative results that can occur for very positive samples, since this preamplification will lower the Cq value of the sample even more. Furthermore, the rtPCR assays will also have a lower cutoff value in the BioMark platform than in the Rotor-Gene Q platform, which also was the case in our study.

The high-throughput rtPCR system can easily be expanded to include more targets since the assay capacity of the 192.24 DA IFC chip used in this study was 24 assays, and thereby an even wider detection system could be developed. However, the added primer-probe sets should be optimized to the temperature conditions selected for the PCR. If more than 24 targets are included, one of the other available DA IFC chips, 48.48 or 96.96, should be utilized. The change in chip format will, however, result in fewer samples that can be analyzed in one run since the choice of chip depends on the application. The high-throughput rtPCR system designed in this study was developed in the frame of a project in which several thousand samples collected from Danish calves were analyzed and, therefore, the 192.24 DA IFC chip was chosen.

The occurrence of pathogens in the field was based on test of pools of pathogen, since it is a much cheaper way of analyzing a large number of animals. One important consideration in the test of pools is to decide on the number of samples to be included in each pool. The schism is to find a balance between the wish to test as many animals as possible and the impact of the number of samples on the sensitivity of the test. Pool size can be theoretically and mathematically calculated (41, 42); however, in real life, this number can be different since pooling of samples have to consider different parameters, such as age and gender. In the present study, both pools consisting of five and 10 individual field samples were examined with the purpose of determining if an acceptable correlation between the results for the individual samples and the pools could be established. Traditionally, when testing pools of samples, it is assumed that each individual sample has the same probability of being positive. However, this is often an erroneous and unrealistic assumption. There exists different pool testing procedures and concluding which one is the best is not an easy task, since parameters such as assay accuracy, availability of risk factor information, prevalence levels, and risk probability distributions all play a role in determining which procedure is best (29, 41, 42). The field samples tested in this study displayed varying Cq values, making it difficult to construct a fully controlled setup. However, the test of pooled samples contra test of individual samples showed that a high-range positive individual sample had a greater influence on the Cq value of the pool than a low-range positive individual sample had, which was observed in all three pool setups. The degree of decrease in the pool's Cq value was affected by either the addition of one or few high-range positive individual sample(s) or by the addition of several mid-range positive individual samples. Whereas the addition of low-range positive individual samples did not noticeably change the Cq value of the pool. This was observed both for the bacteria (*Mycoplasma* spp., *M. bovis, H. somni, T. pyogenes*) and the virus (RVA) in pools consisting of five and 10 individual samples, respectively. Testing pools consisting of a large number of samples can be economically advantageous since it minimize the number of pools. A potential limitation of using larger pools in a group of animals with low occurrence of a given pathogen is the risk of diluting the few positive samples to an extent, that it will no longer be detectable in the rtPCR analysis. However, this study was not able to show how many negative samples were needed to dilute a positive sample so that it was no longer detectable—but again, this depends on the Cq value of the positive sample. Larger pools can nevertheless be preferable in prevalence and screening studies where the purpose often is to test a large number of animals, which also was the case in this study. Since one positive sample in a pool in most cases will lead to a positive test result, the calculation of the occurrence/prevalence of pathogens may be overestimated if it is based on test of pools compared with test of individual animals given the same number of tests performed in each herd.

The high-throughput rtPCR system designed in this study was used to analyze pools of nasal swab and feces field samples in order to determine the occurrence of selected pathogens in Danish calves. The samples were taken from dairy (heifer) and veal (bull) calves at different age groups, and the analysis showed that bacteria were found to be more prevalent than viruses in the nasal swab pools, while it was opposite in the feces pools. The most prevalent respiratory pathogens found in nasal swab pools from the dairy calves were *Mycoplasma* spp., *M. haemolytica, P. multocida*, and *T. pyogenes* (17.5–38.4%), while the other pathogens were observed more sporadically (0–6.9%). All pathogens except for BRSV and *T. pyogenes* were found to be more widespread in the veal calves than in the dairy calves. This was not surprising in that most veal herds commingle with calves from several different sources. Analysis of the occurrence of the pathogens at the herd level revealed that *Mycoplasma* spp., *M. haemolytica*, and *P. multocida* were present in more than 50% of all herds (54.2–94.1%) no matter the herd type. The role of *P. multocida* in the development of pneumonia in calves has been widely discussed, while some studies have reported *P. multocida* as being an opportunistic pathogen, others have found strong indications for *P. multocida* having a pathogenic role (11, 43, 44). Tegtmeier et al. (45) showed that *P. multocida, H. somni, M. haemolytica*, and *T. pyogenes* are among the most common bacteria associated with severe calf pneumonia in Denmark (45). These findings are supported by a newer study, which found *P. multocida, H. somni*, and *M. haemolytica* to be more prevalent in sick cattle than in healthy cattle. Interestingly, >50% of the healthy cattle was found to harbor these bacteria in the lower airways (46), although not showing any symptoms of disease. Benchmarking the laboratory data with information on clinical signs, herd management, housing, and biosecurity may explain why some cattle harboring bacterial pathogens in their lower airways remained healthy, while others developed bronchopneumonia. These analyses are outside the scope of the present study that focused on the establishment and validation of a sensitive and specific system for the detection of pathogens in calves. Nevertheless, the findings in the present study can substantiate that *P. multocida, H. somni, M. haemolytica*, and *T. pyogenes* are present in Danish cattle herds.

In many countries, *M. bovis* is regarded as one of the major causes of respiratory disease in cattle (47). This is supported in the present study, in which the bacterium was detected in 76.5% of the veal herds. Interestingly, this finding is different from older Danish studies, in which *M. bovis* either was not detected in the examined herds or only detected with low occurrence (11, 48, 49). Influenza D virus was detected in four of the veal herds (23.5%), and this is the first time IDV has been found in Danish calves. The virus was isolated for the first time in 2011 in the USA (50), and it has subsequently been detected in cattle from multiple geographic areas across Asia, Europe, and the USA (25, 51–55). In Denmark, BRSV and BCoV have previously been found to be the most common viral agents in relation to calf pneumonia (56). However, BRSV was only detected in very few of the herds (2.4%), while BCoV was found to be more prevalent especially in the veal herds (35.3%). The reason for the low occurrence of the viruses found in the study herds is probably that the herds included in the present study were not tested based on a history of severe respiratory clinical disease which is often the hallmark of especially BRSV.

For the enteric pathogens, RVA was clearly the most prevalent pathogen in the dairy calves (27.4%), while other pathogens were only sporadically detected (0%−1.8%). In the veal calves, RVA (10.9%) and BCoV (12.7%) were the only pathogens detected. Rotavirus A was primary detected in calves below 3 weeks of age, which also was expected since RVA is known to be pathogenic only in young calves (57). Bovine corona virus is also considered an important neonatal calf diarrhea pathogen (58); however, the highest occurrence was detected in veal calves. In contrast, a study from Argentina found BCoV to be most prevalent in the dairy herds (12.1%) compared with veal herds (4.3%) (59). The reason for this discrepancy is probably that only calves with diarrhea was included in the Argentinian study. Another important neonatal diarrhea-causing pathogen is *E. coli* F5, which is known to cause diarrhea within the first 4 days of life (60). In the present study, this pathogen was only detected in the group of 0–10-day-old calves and with a low occurrence (4.8%). This occurrence is similar to the prevalence reported in the Netherlands (2.6%), New Zealand (3.3%), Scotland, and northern England (7.5%) (61–63).

In summary, the developed high-throughput rtPCR system showed good sensitivity and specificity, and the use of it provides new possibilities for more intensive monitoring of bovine respiratory and enteric viral and bacterial pathogens in dairy and veal herds. Furthermore, the system enables testing of multiple samples for the presence of different pathogens in the same setup even with reduced cost and turnover time. Combining the results from continuous monitoring of pathogens with information on clinical signs, productivity, health status, and medicine consumption, the high-throughput rtPCR system presents a new and innovative tool for routine diagnostics, and this even at a lower cost than the traditional diagnostic methods.

## Data Availability Statement

The original contributions presented in the study are included in the article/Supplementary Material, further inquiries can be directed to the corresponding author/s.

## Ethics Statement

Ethical review and approval was not required for the animal study because the samples used in the present study were taken from field animals by veterinarians. The samples were taken and submitted to Centre for Diagnostics, Technical University of Denmark, as if it was a routine submission.

## Author Contributions

LL, BN, MP, and NG contributed to the experimental design of the study. The design of the rtPCR assays used in the present study was done by NG. rtPCR analyses were conducted and interpreted by NG. The manuscript was drafted by NG. LL contributed to the manuscript preparation, while all authors participated in proofreading of the manuscript. All authors read and approved the final manuscript.

## Conflict of Interest

The authors declare that the research was conducted in the absence of any commercial or financial relationships that could be construed as a potential conflict of interest.
