# Supplementary material for: Design of a High-Throughput Real-Time PCR System for Detection of Bovine Respiratory and Enteric Pathogens
Source: Front Vet Sci. 2021 Jun 24;8:677993. doi: 10.3389/fvets.2021.677993 (PMC8267094; doi:10.3389/fvets.2021.677993)
Supplement: Supplementary file 1 [file Table_1.DOCX]

**Supplementary Table 1.** Test of constructed pools with a varying number of individual samples and with a varying number of positive and negative samples.

| **A: *M. bovis*** | | | | | | | | |
| --- | --- | --- | --- | --- | --- | --- | --- | --- |
| **Setup 1** | | | **Setup 2** | | | **Setup 3** | | |
| Pos:Neg | Cq - positive samples | Cq - pools | Pos:Neg | Cq - positive samples | Cq - pools | Pos:Neg | Cq - positive samples | Cq - pools |
| Pool 1:9 | 15.2 | 21.5 | Pool 1:4 | 19.4 | 21.9 | Pool 1:1 | 16.4 | 16.9 |
| Pool 2:8 | 15.2, 21.7 | 22.8 | Pool 2:3 | 19.4, 22.5 | 21.2 | Pool 1:2 | 16.4 | 17.1 |
| Pool 3:7 | 15.2, 21.7, 22.5 | 22.0 | Pool 3:2 | 19.4, 22.5, 13.9 | 15.1 | Pool 1:3 | 16.4 | 17.6 |
| Pool 4:6 | 15.2, 21.7, 22.5, 22.3 | 23.2 | Pool 4:1 | 19.4, 22.5, 13.9, 19.7 | 14.6 | Pool 1:4 | 16.4 | 17.6 |
| Pool 5:5 | 15.2, 21.7, 22.5, 22.3, 16.9 | 20.6 |  |  |  | Pool 1:5 | 16.4 | 18.2 |
| Pool 6:4 | 15.2, 21.7, 22.5, 22.3, 16.9, 20.1 | 20.8 |  |  |  | Pool 1:6 | 16.4 | 18.5 |
| Pool 7:3 | 15.2, 21.7, 22.5, 22.3, 16.9, 20.1, 23.9 | 20.2 |  |  |  | Pool 1:7 | 16.4 | 18.9 |
| Pool 8:2 | 15.2, 21.7, 22.5, 22.3, 16.9, 20.1, 23.9, 23.2 | 19.9 |  |  |  | Pool 1:8 | 16.4 | 19.2 |
| Pool 9:1 | 15.2, 21.7, 22.5, 22.3, 16.9, 20.1, 23.9, 23.2, 21.9 | 19.9 |  |  |  |  |  |  |

| **B: *Mycoplasma* spp.** | | | | | | | | |
| --- | --- | --- | --- | --- | --- | --- | --- | --- |
| **Setup 1** | | | **Setup 2** | | | **Setup 3** | | |
| Pos:Neg | Cq - positive samples | Cq - pools | Pos:Neg | Cq - positive samples | Cq - pools | Pos:Neg | Cq - positive samples | Cq - pools |
| Pool 1:9 | 16.2 | 23.6 | Pool 1:4 | 20.0 | Neg | Pool 1:1 | 16.8 | 18.0 |
| Pool 2:8 | 16.2, 16.8 | 21.8 | Pool 2:3 | 20.0, 24.4 | 21.4 | Pool 1:2 | 16.8 | 18.5 |
| Pool 3:7 | 16.2, 16.8, 22.2 | 21.7 | Pool 3:2 | 20.0, 24.4, 15.1 | 17.0 | Pool 1:3 | 16.8 | 19.4 |
| Pool 4:6 | 16.2, 16.8, 22.2, 20.5 | 21.5 | Pool 4:1 | 20.0, 24.4, 15.1, 20.5 | 16.1 | Pool 1:4 | 16.8 | 18.9 |
| Pool 5:5 | 16.2, 16.8, 22.2, 20.5, 16.0 | 19.9 |  |  |  | Pool 1:5 | 16.8 | 19.3 |
| Pool 6:4 | 16.2, 16.8, 22.2, 20.5, 16.0, 21.0 | 20.4 |  |  |  | Pool 1:6 | 16.8 | 20.0 |
| Pool 7:3 | 16.2, 16.8, 22.2, 20.5, 16.0, 21.0, 19.5 | 19.9 |  |  |  | Pool 1:7 | 16.8 | 20.9 |
| Pool 8:2 | 16.2, 16.8, 22.2, 20.5, 16.0, 21.0, 19.5, 17.5 | 19.0 |  |  |  | Pool 1:8 | 16.8 | 20.1 |
| Pool 9:1 | 16.2, 16.8, 22.2, 20.5, 16.0, 21.0, 19.5, 17.5, 15.1 | 18.3 |  |  |  |  |  |  |

| **C: *H. somni*** | | | | | | | | |
| --- | --- | --- | --- | --- | --- | --- | --- | --- |
| **Setup 1** | | | **Setup 2** | | | **Setup 3** | | |
| Pos:Neg | Cq - positive samples | Cq - pools | Pos:Neg | Cq - positive samples | Cq - pools | Pos:Neg | Cq - positive samples | Cq - pools |
| Pool 1:9 | 19.9 | 22.9 | Pool 1:4 | 16.4 | 17.9 | Pool 1:1 | 17.9 | 17.5 |
| Pool 2:8 | 19.9, 17.9 | 20.9 | Pool 2:3 | 16.4, 22.7 | 17.7 | Pool 1:2 | 17.9 | 17.9 |
| Pool 3:7 | 19.9, 17.9, 17.4 | 20.0 | Pool 3:2 | 16.4, 22.7, 22.2 | 17.0 | Pool 1:3 | 17.9 | 18.9 |
| Pool 4:6 | 19.9, 17.9, 17.4, 15.9 | 17.0 | Pool 4:1 | 16.4, 22.7, 22.2, 22.1 | 17.1 | Pool 1:4 | 17.9 | 18.4 |
| Pool 5:5 | 19.9, 17.9, 17.4, 15.9, 18.8 | 16.2 |  |  |  | Pool 1:5 | 17.9 | 19.1 |
| Pool 6:4 | 19.9, 17.9, 17.4, 15.9, 18.8, 20.5 | 16.9 |  |  |  | Pool 1:6 | 17.9 | 19.0 |
| Pool 7:3 | 19.9, 17.9, 17.4, 15.9, 18.8, 20.5, 20,3 | 16.4 |  |  |  | Pool 1:7 | 17.9 | 19.8 |
| Pool 8:2 | 19.9, 17.9, 17.4, 15.9, 18.8, 20.5, 20,3, 17.8 | 16.1 |  |  |  | Pool 1:8 | 17.9 | 19.6 |
| Pool 9:1 | 19.9, 17.9, 17.4, 15.9, 18.8, 20.5, 20,3, 17.8, 18.9 | 16.6 |  |  |  |  |  |  |

| **D: *T. pyogenes*** | | | | | | | | |
| --- | --- | --- | --- | --- | --- | --- | --- | --- |
| **Setup 1** | | | **Setup 2** | | | **Setup 3** | | |
| Pos:Neg | Cq - positive samples | Cq - pools | Pos:Neg | Cq - positive samples | Cq - pools | Pos:Neg | Cq - positive samples | Cq - pools |
| Pool 1:9 | 16.3 | 17.9 | Pool 1:4 | 22.5 | 23.1 | Pool 1:1 | 17.0 | 17.5 |
| Pool 2:8 | 16.3, 22.2 | 18.3 | Pool 2:3 | 22.5, 18.6 | 21.0 | Pool 1:2 | 17.0 | 17.8 |
| Pool 3:7 | 16.3, 22.2, 22.4 | 18.1 | Pool 3:2 | 22.5, 18.6, 21.4 | 21.7 | Pool 1:3 | 17.0 | 18.3 |
| Pool 4:6 | 16.3, 22.2, 22.4, 21.1 | 17.7 | Pool 4:1 | 22.5, 18.6, 21.4, 24.0 | 20.6 | Pool 1:4 | 17.0 | 18.1 |
| Pool 5:5 | 16.3, 22.2, 22.4, 21.1, 22.3 | 17.9 |  |  |  | Pool 1:5 | 17.0 | 18.6 |
| Pool 6:4 | 16.3, 22.2, 22.4, 21.1, 22.3, 21.5 | 17.7 |  |  |  | Pool 1:6 | 17.0 | 17.9 |
| Pool 7:3 | 16.3, 22.2, 22.4, 21.1, 22.3, 21.5, 20.6 | 18.5 |  |  |  | Pool 1:7 | 17.0 | 18.1 |
| Pool 8:2 | 16.3, 22.2, 22.4, 21.1, 22.3, 21.5, 20.6, 22.4 | 18.3 |  |  |  | Pool 1:8 | 17.0 | 18.4 |
| Pool 9:1 | 16.3, 22.2, 22.4, 21.1, 22.3, 21.5, 20.6, 22.4, 24.1 | 17.6 |  |  |  |  |  |  |

| **E: Rotavirus A** | | | | | | | | |
| --- | --- | --- | --- | --- | --- | --- | --- | --- |
| **Setup 1** | | | **Setup 2** | | | **Setup 3** | | |
| Pos:Neg | Cq - positive samples | Cq - pools | Pos:Neg | Cq - positive samples | Cq - pools | Pos:Neg | Cq - positive samples | Cq - pools |
| Pool 1:9 | 11.8 | 20.7 | Pool 1:4 | 10.6 | 17.2 | Pool 1:1 | 8.3 | 8.8 |
| Pool 2:8 | 11.8, 10.9 | 17.1 | Pool 2:3 | 10.6, 7.7 | 12.1 | Pool 1:2 | 8.3 | 9.1 |
| Pool 3:7 | 11.8, 10.9, 7.8 | 16.6 | Pool 3:2 | 10.6, 7.7, 13.7 | 11.9 | Pool 1:3 | 8.3 | 12.6 |
| Pool 4:6 | 11.8, 10.9, 7.8, 10.7 | 16.5 | Pool 4:1 | 10.6, 7.7, 13.7, 7.3 | 11.1 | Pool 1:4 | 8.3 | 14.9 |
| Pool 5:5 | 11.8, 10.9, 7.8, 10.7, 19.1 | 12.5 |  |  |  | Pool 1:5 | 8.3 | 14.9 |
| Pool 6:4 | 11.8, 10.9, 7.8, 10.7, 19.1, 7.1 | 11.5 |  |  |  | Pool 1:6 | 8.3 | 15.6 |
| Pool 7:3 | 11.8, 10.9, 7.8, 10.7, 19.1, 7.1, 17.8 | 17.2 |  |  |  | Pool 1:7 | 8.3 | 15.4 |
| Pool 8:2 | 11.8, 10.9, 7.8, 10.7, 19.1, 7.1, 17.8, 16.0 | 12.8 |  |  |  | Pool 1:8 | 8.3 | 16.4 |
| Pool 9:1 | 11.8, 10.9, 7.8, 10.7, 19.1, 7.1, 17.8, 16.0, 9.1 | 14.3 |  |  |  |  |  |  |
